# Anorexia nervosa manifesting as massive ascites, hypercholesterolemia, and sequential binge eating in an 11-year-old girl: A case report

**DOI:** 10.1097/MD.0000000000021739

**Published:** 2020-08-28

**Authors:** Hung-Hao Fan, I-Cheng Lin, Jing-Er Chen, Wei-Hua Lee, Shiuh-Bin Fang

**Affiliations:** a Division of Pediatric Gastroenterology, Hepatology, and Nutrition, Department of Pediatrics; b Department of Pediatrics, School of Medicine, College of Medicine; c Department of Psychiatry; d Department of Pathology, Shuang Ho Hospital; e Master Program for Clinical Pharmacogenomics and Pharmacoproteomics, College of Pharmacy, Taipei Medical University, Taipei, Taiwan.

**Keywords:** anorexia nervosa, binge eating, hypercholesterolemia, hypocomplementemia, massive ascites

## Abstract

**Rationale:**

Anorexia nervosa (AN) is a serious eating disorder associated with a distorted body image. Hypercholesterolemia has been found in patients with AN but the mechanism of hyperlipidemia in AN remains little known. Ascites in patients with AN has been attributed to hypoalbuminemia and liver diseases, but massive ascites without the aforementioned etiologies has never been reported in AN.

**Patient concerns:**

An 11-year-old girl was admitted for exclusion of organic underlying diseases due to severe body weight loss (18% within 3 weeks), poor appetite, and hypercholesterolemia (274 mg/dL). She complained of heartburn sensation, nausea, vomiting, constipation, and postprandial dull abdominal pain with fullness.

**Diagnoses:**

The patient's condition met with all 3 of the Diagnostic and Statistical Manual of Mental Disorders (DSM-5) criteria for diagnosing AN. On admission, her total cholesterol level was 337 mg/dL and hypocomplementemia (C3 55.5 mg/dL) was also found. Abdominal sonography and computed tomography scans showed massive ascites. However, neither proteinuria nor hypoalbuminemia was found. Upper gastroduodenal endoscopy showed chronic superficial gastritis and colonoscopy revealed negative findings. Ascites obtained by paracentesis demonstrated a transudate without bacterial infection, tuberculosis, or pancreatitis. Exploratory laparoscopy showed nonpurulent ascites. However, biopsies from the small intestine, mesentery, and liver showed chronic inflammation and fibrosis.

**Interventions:**

The intensive nutritional therapy by increasing total energy intake stepwise with a combination of high-energy formula and her favorite foods.

**Outcomes:**

Her hypercholesterolemia, hypocomplementemia, and massive ascites resolved after her weight was restored. She developed binge eating with continuous weight gain after discharge. Her weight significantly increased to an obese level (body mass index [BMI] 25.9 kg/m^2^) after loss to follow-up for 4 years until she returned to our emergency room due to suicide attempt.

**Conclusion:**

Diagnostic crossover between subtypes in anorexia nervosa might be a potential risk factor for illness severity and poor prognosis. AN can manifest as massive ascites with normal albumin concentrations that could possibly be due to chronic inflammation of the intestinal serosa, mesentery, and peritoneal surface of the liver.

## 1 Introduction

Anorexia nervosa (AN) and bulimia nervosa (BN) are serious psychiatric diseases associated with disordered eating and a distorted body image with considerable morbidity and mortality.^[1]^ Hypercholesterolemia has been found in patients with AN since 1965.^[2]^ Additionally, ascites in patients with AN has been attributed to hypoalbuminemia and liver diseases.^[3,4]^ However, massive ascites without the aforementioned etiologies has never been reported in AN.

## 2 Case description

The Taipei Medical University-Joint Institutional Review Board (TMU-JIRB) approved waiver of the written informed consent from the participants’ legal guardian/next of kin (Approval No. N201601013) after the patient and her mother had been informed by telephone for publishing the case report but lost-to-follow up. We reported to the TMU-JIRB committee for thorough evaluation for publishing the case report under full protection of the patient's privacy and right.

An 11-year-old girl, a fifth grade elementary school student, was admitted due to 18% (34–28 kg) body weight loss within 3 weeks, poor appetite, and hypercholesterolemia (274 mg/dL) at a local clinic. She had been performing diet control since age 8 as she was being teased by her friends for her obesity (135 cm, 55 kg, BMI 30.2 kg/m^2^). She started to lose weight after restricting her food intake, avoiding high-calorie foods, and engaging in intensive exercise. One year later, her weight decreased to 40 kg and severe constipation developed. She also complained of postprandial dull abdominal pain with heartburn sensation and fullness, nausea, and vomiting for months. She was afraid of eating foods, became irritable and impulsive, and her oral intake reduced considerably.

She was brought to our psychologist's clinic (34 kg, 145 cm, BMI 16.2 kg/m^2^). She was highly self-disciplined with both school performance and restricted caloric intake (<500 kcal/d) and presented with hypoglycemia (glucose 50 mg/dL), hyperphosphatemia (phosphate 4.9 mg/dL), and hypocapnia (carbon dioxide 19 mEq/L). AN was diagnosed according to the Diagnostic and Statistical Manual of Mental Disorders (DSM-5) criteria,^[5]^ and she was referred to our pediatric gastroenterologist's clinic for further work-up to exclude organic diseases. On admission 3 weeks after her visit to the psychologist's clinic, she appeared emaciated with a weight of 28 kg (10th–15th percentile), a height of 145 cm (50th–60th percentile), and a BMI of 13.3 kg/m^2^. The cachexic girl had stable vital signs (body temperature 36 °C, respiratory rate 19 breaths/min, and blood pressure 91/60 mmHg), except for tachycardia (96 beats/min). On initial physical examination, she had poor skin turgor, a soft and flat abdomen with localized tenderness in the epigastric and periumbilical areas, and shifting dullness. The rest of the physical examination showed normal results, with neither hepatosplenomegaly nor pitting edema. Her complete blood cell count revealed a leukocyte count of 6000/mm^3^ with 43% neutrophils and 50% lymphocytes, a platelet count of 272 × 10^3^/mm^3^, hemoglobin of 11.6 mg/dL, and hematocrit of 35.1%. Her serum chemistries, including aspartate transaminase (37 IU/L), alanine transaminase (24 IU/L), alkaline phosphatase (38 IU/L), γ-glutamyl transpeptidase (14 IU/L), albumin (3.5 g/dL), amylase (25 U/L), blood urea nitrogen (20 mg/dL), and creatinine (0.6 mg/dL), was within the normal range, whereas her urine and stool routines were negative.

**Figure 1:**
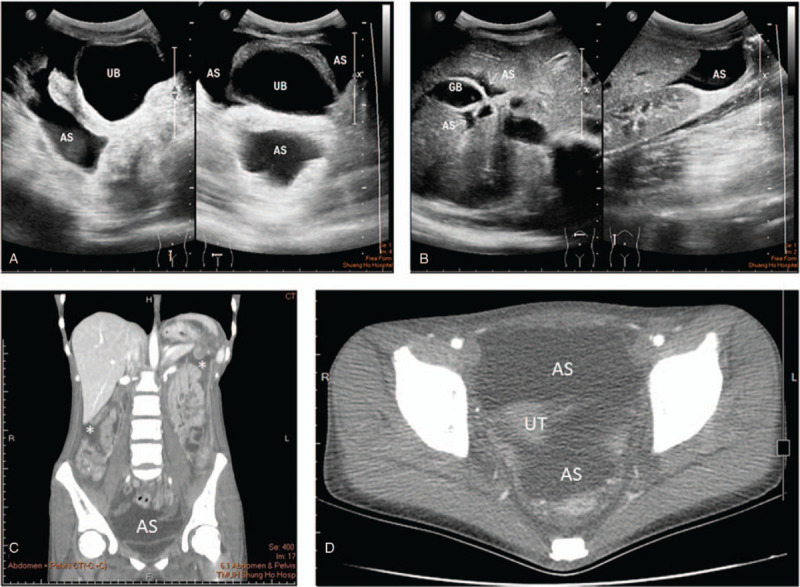
Abdominal sonography and CT scan showed diffuse ascites in the pelvis (AS in A and C: coronal section), Morison pouch, and subsplenic space (AS in B and asterisks in D: transverse section). AS = ascites, GB = gallbladder, UB = urinary bladder, UT = uterus.

**Figure 2:**
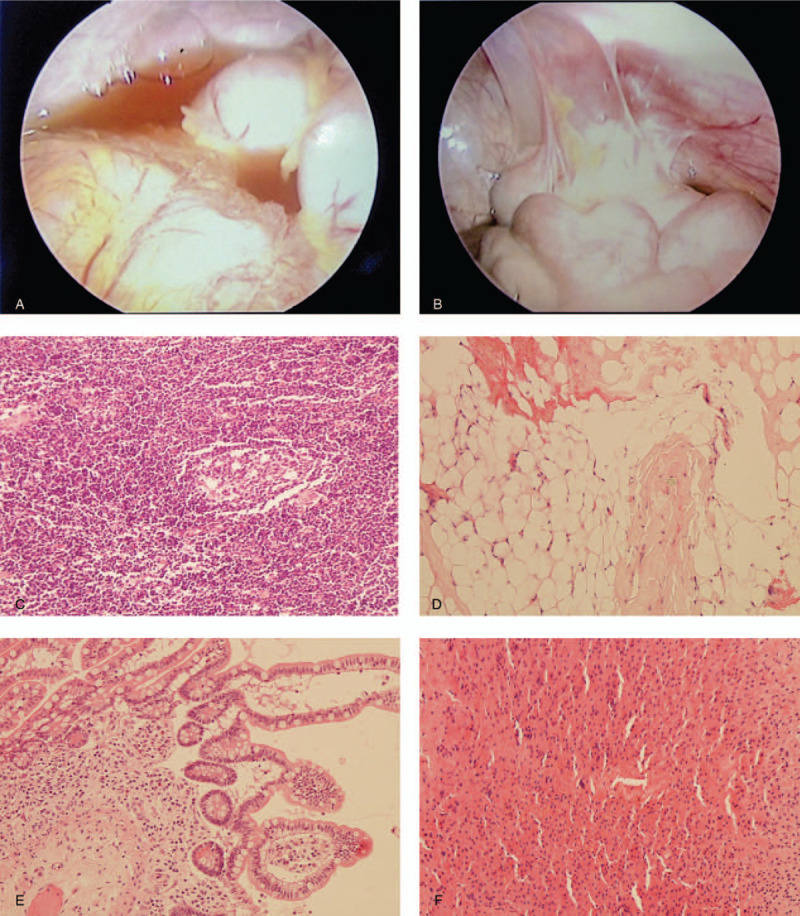
Laparoscopy of the peritoneal cavity showed nonpurulent ascites and a mildly hyperemic edematous peritoneum without any mass lesion (A, B). Histopathology by hematoxylin and eosin staining (magnification power: 200×) of the laparoscopic biopsies of mesenteric lymph nodes (C), the mesentery (D), the small intestine (E), and the liver (F).

Abdominal sonography and computed tomography (CT) scans showed massive ascites and suspicious colitis involving descending to the sigmoid colon (Fig. 1A–D). However, neither proteinuria nor hypoalbuminemia (albumin 3.5 g/dL on Day 5 and 3.9 g/dL on Day 8) was found. Laboratory tests for autoimmune diseases, including ANA, anti-dsDNA, and direct and indirect Coombs tests, were negative. Thyroid function (thyroid-stimulating hormone 1.91 μIU/mL, free T4 0.73 ng/dL) was within normal ranges. The girl's cholesterol level was 337 mg/dL, with a high low-density lipoprotein-cholesterol (LDL-C) level of 241 mg/dL (<130) and a normal high-density lipoprotein-cholesterol (HDL-C) level of 63 mg/dL (>40). Her triglyceride level was 56 mg/dL (<150). Hypocomplementemia (C3 55.5 mg/dL [90–180]), decreased total iron-binding capacity (177 μg/dL [260–445]), and an elevated ferritin level (518.6 ng/dL [11–307]) were also found, but her serum iron concentration was normal (70 μg/dL [28–170]). Tumor markers, including α-fetoprotein, β-human chorionic gonadotropin, CA125, CA199, and carcinoembryonic antigen, were all within normal ranges. Upper gastroduodenal endoscopy and pathology of biopsies showed chronic superficial gastritis, but colonoscopy revealed negative findings except for hemorrhoids. Paracentesis for ascitic fluid analysis demonstrated a transudate without bacterial infection, tuberculosis, or pancreatitis, and ascites cytology showed no malignant cells. Ascitic fluid routine, including leukocytes (10 cells/μL), erythrocytes (3 cells/μL), negative Gram staining, glucose (95 mg/dL), total protein (1.4 g/dL), albumin (1.9 g/dL), amylase (25 U/L), and lactate dehydrogenase (26 IU/L), was within the normal range. Ascitic fluid for aerobic and anaerobic cultures, tuberculosis culture, and fungus culture grew no bacteria. Exploratory laparoscopy showed nonpurulent ascites with mildly hyperemic edematous peritoneum (Fig. 2A and B). However, laparoscopic biopsies showed histopathologically lymphoid hyperplasia in the mesenteric lymph nodes, chronic erosive inflammation in the small intestine, fatty tissues with fibrosis in the mesentery, and reactive change with mild cholestasis in the liver (Fig. 2C–F).

The initial goal for daily total energy intake was set at 1000 kcal/d and increased stepwise to 1800 to 2000 kcal/d to help the girl achieve an ideal body weight of 33 to 44 kg. However, she kept walking around the ward during hospitalization and nasogastric intubation failed. A combination of high-energy formula and her favorite foods were administered. Pitting edema in the bilateral lower limbs developed on the 8th hospital day but disappeared 3 days later. Her weight increased to 32.3 kg on the 6th day, and she was discharged on the 15th day. After discharge, she began recurrent binge eating with compensatory behaviors to prevent weight gain (i.e., excessive exercise and self-evaluation influenced by body shape and weight). Nevertheless, 19 days after discharge, her weight increased to 35 kg, and 49 days later, it increased to 40 kg. Her repeated levels of cholesterol (178 mg/dL), LDL-C (71 mg/dL), C3 (100 g/dL), and alkaline phosphatase (68 IU/L) were normalized 39 days after discharge. Follow-up abdominal sonography showed massive ascites, but these disappeared 49 days after discharge. Unfortunately, she was lost to follow-up for 4 years until she was sent to our emergency room to get her cut wrist sutured following a suicide attempt. By that time, she had become obese (weight 60 kg, height 152 cm, BMI 25.9 kg/m^2^).

## 3 Discussion

Our patient's clinical manifestations met with all 3 of the DSM-5 criteria for diagnosing AN.^[5]^ In addition, edema or fluid retention is a common complication in patients with AN. Refeeding syndrome develops when the nutritional state is corrected rapidly and is defined by 3 critical facets: peripheral edema or acute circulatory fluid overload, disturbance to organ function, and severely low electrolyte concentrations.^[6]^ However, our patient manifested peripheral edema after refeeding transiently for 3 days without the other 2 facets and improved after energy supplementation was decelerated.

Our case developed hypercholesterolemia and high LDL-C levels, which have been found ranging between 14.5% and 76% in patients with AN.^[7–11]^ Patients with AN of the binge eating/purging-type (AN-B) exhibit higher concentrations of total cholesterol (TC) and LDL-C than do patients with bulimia nervosa (BN) and controls, as well as higher triglyceride (TG) levels than do controls (Case T 1999). Our case with AN with restrictive and binge eating had similarly high levels of TC and LDL-C to patients with AN-B, but her normal TG level was more similar to patients with BN. In addition, more than half of patients with AN prospectively develop bulimic symptoms, crossing over to the binge/purge subtype of AN or to BN, and one-third of those with BN have a history of AN.^[12]^ In 23 women with AN, 5 patients all had hypercholesterolemia (mean 239.6 mg/dL, range 218–264 mg/dL) with the high levels of LDL-C (mean 149.4 mg/dL, range 136–173 mg/dL) but an normal average HDL-C level (mean 54.2 mg/dL, range 32–80 mg/dL).^[13]^ Similarly, our case showed high levels of TC and LDL and a normal HDL-C level. The mechanism of hyperlipidemia in AN and why the levels of serum lipids vary among different types of eating disorders remains unknown. So far, several assumptions have been hypothesized. Elevated TC levels in AN might be caused by high LDL-C levels, mostly determined by loss of body fat and the subsequent alternation in thyroid hormones, which is a result of decreased TC catabolism due to changed LDL receptor activity.^[14]^ The higher TC levels are attributed to high LDL-C levels,^[9]^ but this is not confirmed.^[13]^ Possible mechanisms for hypercholesterolemia in AN include reduced cholesterol and bile acid turnover, a starvation-induced flux of peripheral cholesterol to the liver, downregulated cholesterol synthesis in the liver, delayed catabolism of LDL lipoproteins due to reduced LDL receptor activity of the liver, and decreased triiodothyronine concentration.^[10]^ During starvation, thyroxin activity can be decreased and cholesterol turnover can be increased due to accelerated cholesterol ester transfer protein (CETP) activity. This allows the body to maintain its cholesterol level by releasing cholesterol from cells when cholesterol is lacking from foods. Start of refeeding and recovery from AN lead to normalization of the lipid profile, CETP, and apolipoproteins.^[10,11,14]^ However, the normal thyroid hormone level in our case suggests another pathophysiology without the involvement of thyroxin.

Idiopathic massive ascites is rarely seen in patients with AN without hypoalbuminemia. Moderate ascites because of acute liver cell damage was observed in 4 of 12 patients with AN.^[3]^ Marked ascites has been reported in 3 cases of AN with hypoalbuminemia, including one developing massive ascites and moderate pedal edema with elevated CA-125,^[4]^ another developing abdominal pain and ascites due to chronic pancreatitis,^[15]^ and 2 of 3 women with AN developing portal hypertension and gastroesophageal varices but pathologically mild pericellular fibrosis.^[16]^ In our case, the aforementioned common causes for massive ascites in AN were not found.

Complements play an important role in host immunity and some are produced in adipose tissue. Complement levels of C3, Factor B, Factor D, and AP50 in the alternative pathway are low in starving anorectics and can be normalized with weight gain.^[17]^ Complement C3 serum levels correlate with BMI and can serve as a sensitive biomarker for monitoring the severity of disease in AN.^[18]^ Similarly, our case manifested the concordant result in AN with a low C3 serum level that was normalized after gaining weight.

Both AN and BN are serious psychiatric diseases associated with disordered eating and a distorted body image with considerable morbidity and mortality.^[1]^ According to the DSM-5 criteria, AN is defined by low weight and may or may not involve binge eating or compensatory behaviors, whereas the hallmarks of BN are recurrent binge eating and compensatory behaviors at least once a week for 3 months in individuals who are typically of normal weight.^[5]^ However, crossover between AN and BN has been reported at approximately 8% to 54% from AN to BN and approximately 4% to 27% from BN to AN.^[19,20]^ Although both AN and BN are chronic and relapsing conditions, a 9-year longitudinal follow-up study indicated that women with BN who had a history of AN are more likely to have a protracted illness, relapsing into AN during follow-up. The same study also suggested that AN carries a poorer prognosis compared with BN in terms of time to and likelihood of full recovery as well as increased risk of mortality.^[12]^ Limited by the lost to follow-up in our case, whether our patient developed recurrent inappropriate compensatory behavior to prevent weight gain for >3 months to meet with the diagnostic criteria of BN is unknown but possible.

## 4 Conclusion

We described an unusual case of AN in an 11-year-old girl manifesting with severe body weight loss, marked ascites, and hypercholesterolemia with high LDL-C and normal HDL-C levels. After treatment, her ascites resolved with weight recovery in parallel with normalized levels of TC and C3, but she developed binge eating after discharge and even attempted suicide with failure 4 years later, validating diagnostic crossover between subtypes in AN, or possibly BN, as a potential risk factor for illness severity and poor prognosis. To our knowledge, this is the first case report of AN manifesting as massive ascites without hypoalbuminemia, probably due to chronic inflammation of the intestinal serosa, mesentery, and peritoneal surface of the liver.

## Acknowledgment

The authors thank Dr Jun-Yu Kao, Dr Shu-Huey Chen, Dr Yung-Ting Kuo, Dr Wei-Hsuan Liao, Dr Jenn-Ming Yang, and Dr Shu-Hung Wang for their expertise and assistance in the patient care.

## Author contributions

**Conceptualization:** Shiuh-Bin Fang.

**Data curation:** Hung-Hao Fan, I-Cheng Lin, Jing-Er Chen, Wei-Hua Lee.

**Investigation:** Hung-Hao Fan, I-Cheng Lin.

**Supervision:** Shiuh-Bin Fang.

**Visualization:** Hung-Hao Fan, Jing-Er Chen, Wei-Hua Lee.

**Writing – original draft:** Hung-Hao Fan.

**Writing – review & editing:** I-Cheng Lin, Shiuh-Bin Fang.
